# A Study on Establishing a Microstructure-Related Hardness Model with Precipitate Segmentation Using Deep Learning Method

**DOI:** 10.3390/ma13051256

**Published:** 2020-03-10

**Authors:** Chan Wang, Duoqi Shi, Shaolin Li

**Affiliations:** 1 School of Energy and Power Engineering, Beihang University, Beijing 100191, China; wangchan1204@126.com; 2 Collaborative Innovation Center of Advanced Aero–Engine, Beijing 100191, China

**Keywords:** deep learning method, different generations of γ’ precipitates, large-area SEM images, γ’ coarsening, microstructure-related hardness model

## Abstract

This paper established a microstructure-related hardness model of a polycrystalline Ni-based superalloy GH4720Li, and the sizes and area fractions of γ’ precipitates were extracted from scanning electron microscope (SEM) images using a deep learning method. The common method used to obtain morphological parameters of γ’ precipitates is the thresholding method. However, this method is not suitable for distinguishing different generations of γ’ precipitates with similar gray values in SEM images, which needs many manual interventions. In this paper, we employ SEM with ATLAS (AuTomated Large Area Scanning) module to automatically and quickly detect a much wider range of microstructures. A deep learning method of U-Net is firstly applied to automatically and accurately segment different generations of γ’ precipitates and extract their parameters from the large-area SEM images. Then the obtained sizes and area fractions of γ’ precipitates are used to study the precipitate stability and microstructure-related hardness of GH4720Li alloy at long-term service temperatures. The experimental results show that primary and secondary γ’ precipitates show good stability under long-term service temperatures. Tertiary γ’ precipitates coarsen selectively, and their coarsening behavior can be predicted by the Lifshitz–Slyozov encounter modified (LSEM) model. The hardness decreases as a result of γ’ coarsening. A microstructure-related hardness model for correlating the hardness of the γ’/γ coherent structures and the microstructure is established, which can effectively predict the hardness of the alloy with different microstructures.

## 1. Introduction

The strength of Ni_3_Al-based alloy is primarily derived from the coherent L1_2_-γ’ precipitates, which are embedded in a face-centered cubic (fcc) γ-Ni matrix. The morphology and distribution of γ’ precipitates have a significant impact on the mechanical properties and hardness of Ni-based superalloys [1,2,3,4,5]. As documented in numerous studies, γ’ precipitates as a main microstructural characteristic of Ni-based superalloys are not stable at elevated temperatures [6,7,8,9]. Smaller γ’ precipitates with a large surface-area-to-volume ratio will grow to a smaller number of larger γ’ precipitates at high temperatures, in order to decrease the total energy of the system by decreasing interfacial energy. This process is realized by the solute diffusion, and this diffusion process is referred to as coarsening or Ostwald ripening [10,11]. With increasing the service temperature and time of superalloys, some neighboring γ’ precipitates even coalesce to further decrease the total energy of system [12].

A polycrystalline Ni-based superalloy GH4720Li, with good high-temperature strength [13], has been extensively applied to make aero-engine turbine disks working at temperatures of about 600 °C–750 °C. In this alloy, multiple generations of γ’ precipitates can be formed during heat treatment with slow cooling rates, including the primary, secondary and tertiary γ’ precipitates. The largest primary γ’ precipitates are present when precipitates are not completely dissolved during the subsolvus solution heat treatment. The secondary and tertiary γ’ precipitates are formed by two nucleation bursts during slow cooling [14]. The γ’ coarsening behaviors in this alloy have been studied extensively, and many quantitative studies have been performed during various heat treatment processes [15,16]. The γ’ precipitates in this alloy will be largely dissolved during solution treatment at temperatures above 1140 °C. Their average size increases and volume fraction decrease with increasing the solutioning temperature. The size and morphology of secondary γ’ precipitates can be significantly influenced by the cooling rate after the solution treatment. The size of secondary γ’ precipitates increases significantly with decreasing the cooling rate, and the morphology changes from fine spherical particles to large irregular-shaped particles with slower cooling rates. The size of tertiary γ’ precipitates increases during aging. However, there is few quantitative data on the precipitate stability of GH4720Li alloy at service temperatures for a long time. For Ni-based superalloys, the γ’ precipitates strengthen the hardness of alloys, and the morphology and distribution of γ’ precipitates have significant effects on the hardness [17,18,19]. The depletion and coarsening of γ’ precipitates decrease the hardness of alloys, whereas the recovery and recrystallization increasing the number of the γ’ precipitates increase the alloy hardness. However, there is no hardness model which could correlate the hardness and the γ’ morphology, in order to predict the hardness of Ni-based superalloys with different microstructures.

Precise recognition and segmentation of γ’ precipitates from large quantities of scanning electron microscope (SEM) images is the most important step for extracting morphological parameters of γ’ precipitates to quantitatively study the precipitate stability and the related properties of alloys. The common approach to segment γ’ precipitates is simple thresholding, which is effective when there have obvious differences in the gray value between target and background. The quantitative parameters of γ’ precipitates in Ni-based superalloys are obtained mostly by this approach. However, this approach is not suitable for distinguishing different generations of γ’ precipitates in Ni-based superalloys, which have similar gray values. It needs a lot of manual interventions during thresholding [10], resulting in unpredicted errors. Furthermore, when there are too many SEM images or each SEM image contains too much information, this work will take a very long time and a high labor cost. Other experimental methods can also be applied to distinguish different phases of alloys based on the difference of their compositions or structures in order to obtain the sizes or volume fractions of the phases, such as EDX elemental mapping [20], X-ray diffraction (XRD). But these experimental methods will take a high cost and the results are not accurate. Moreover, these methods are also difficult to distinguish different generations of γ’ precipitates which are the same phase with different size distributions.

Deep learning has become a strong tool for automatic image segmentation and achieved the state-of-the-art results. The convolutional neural networks (CNNs) use relatively little pre-processing and automatically learn representative complex features directly from the data itself. Therefore, CNNs have been widely applied to segment the various medical images [21,22]. However, CNNs have size requirement for the input images, and the calculation is inefficient. Jonathan Long et al. [23] proposed the fully convolutional networks (FCNs), which were first trained end-to-end, pixels-to-pixels on semantic segmentation. Moreover, FCNs can be used to segment arbitrarily large images. Olaf Ronneberger et al. [24] modified and extended the architecture of fully convolutional network and proposed a U-Net convolutional neural network (U-Net). This strategy also allows the seamless segmentation of arbitrarily large images. Furthermore, this strategy uses very few training images and yields more precise segmentations.

In this study, γ’ precipitate stability and hardness of GH4720Li alloy are investigated in the temperature range from 630 °C to 760 °C for 500–2500 h. The experimental temperatures are determined based on the service temperature of the turbine disk. It is very difficult to accurately and quickly segment different generations of γ’ precipitates from massive SEM images, and the problem has not been solved properly in open literatures. In this paper, a new strategy is proposed to apply learning method to segment γ’ precipitates from the extremely large-area SEM images and then obtain their sizes and area fractions. Based on the obtained data, a microstructure-related hardness model is established, which can correlate the microstructure and the hardness of the γ’/γ coherent structures of Ni-based alloys. To the authors’ knowledge, it is the first time to employ a deep learning algorithm to automatically segment γ’ precipitates and extract their parameters to quantitative research, which could greatly promote the application of deep learning in materials science.

## 2. Materials and Experimental Methods

The cuboid-shaped samples of GH4720Li superalloy with size of 30 mm × 5 mm × 5 mm were used for thermal exposure tests, and the experimental parameters are shown in Table 1. The microstructure of sample at initial condition was characterized as the data at time 0 h of the thermal exposure tests. After the tests, the cuboid samples at initial condition and after different tests were grinded and mechanically polished, then they were electro-etched by a solution of 15 g CrO_3_ + 10 mL H_2_SO_4_ + 150 mL H_3_PO_4_ with voltage of 5 V to emerge the γ’ precipitates of the alloy. Subsequently, the γ’ evolution was observed by Zeiss SUPRA55 field emission scanning electron microscope (Carl Zeiss, Jena, Germany) with ATLAS (AuTomated Large Area Scanning, Carl Zeiss, Jena, (TH), Germany) integrated module, which can be used to automatically and quickly detect a wider range of microstructures and produce high-resolution large area images. Because of the inhomogeneous distribution of primary and secondary γ’ precipitates in GH4720Li alloy, the large-area SEM images could ensure the statistical accuracy of parameters. The pixel size of each large-area SEM image is 10 nm. For the digital images, the number of pixels is usually expressed in units of K (1024 pixels) [25]. The area of each SEM image is 32 K pixels × 32 K pixels, corresponding to 327.68 μm × 327.68 μm. Each large-area SEM image contains at least 2300 primary γ’ precipitates and at least 3500 secondary γ’ precipitates. In addition, some small SEM images are used in this paper to show the γ’ morphologies under different test conditions.

After the different thermal exposure tests, the nanoindentation experiments were performed to test the nanoindentation hardness of the γ’/γ coherent structure inside grains of the alloy. The nanoindentation experiments were carried out using a Bruker UMT-2 nanoindenter (Bruker Nano Inc., Campbell, CA, USA) with a load force of 50 mN. Each sample was tested 5 times at different positions of samples, and all indentations were made in the interior of grains. The effect of carbides can be avoided by selecting proper test positions.

## 3. Segmentation of γ’ Precipitates Using U-Net

The U-Net model [24] is employed to recognize and segment different generations of γ’ precipitates from 2D SEM images. Then the morphological parameters of each γ’ precipitate including diameter *d* and area *A_j_* are calculated in order to quantitatively describe the γ’ morphology during thermal exposure. The equivalent circular-area diameter is calculated as the diameter *d* of γ’ precipitate [26]. Finally, the average diameter $\bar{d}$ and the area fraction $\bar{f}$ of different generations of γ’ precipitates are calculated respectively using Equations (1) and (2).

(1) $$\bar{d}=\frac{1}{N}\sum_{j=1}^{N}(2\times \sqrt{\frac{A_{j}}{\pi })}$$

(2) $$\bar{f}=\frac{\sum_{j=1}^{N}A_{j}}{A}$$

where *N* is the number of one kind of γ’ precipitates in the large-area SEM image. *A_j_* is the area of the *j*-th γ’ precipitate. *A* is the area of the large-area SEM image.

**Figure 1:**
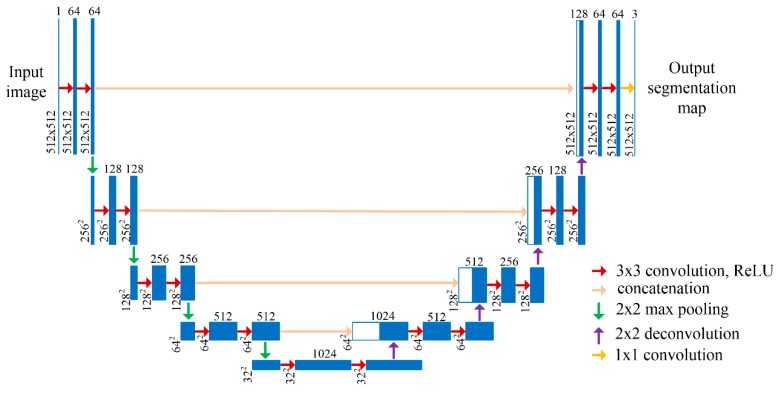
U-net architecture.

The U-Net architecture consists of a contracting path and an expansive path as shown in Figure 1. There are 23 convolutional layers in the U-Net network. The contracting path is used to extract a hierarchy of increasingly complex features from input images. The contracting path consists of the repeated application of two 3 × 3 convolutions with stride 1, each followed by a ReLU activation function and a 2 × 2 max pooling operation with stride 2 for downsampling. The number of feature channels is doubled at each downsampling step. The expansive path takes the feature representation and recovers the feature maps to the input dimensions, realizing pixel prediction in the original image. The expansive path consists of the repeated application of a 2 × 2 deconvolution which upsamples the feature maps and halves the number of feature channels, a concatenation with the correspondingly cropped feature map from the contracting path to improve the prediction accuracy, and two 3 × 3 convolutions, each followed by a ReLU. Then 64-component feature vectors can be produced after repeating these steps 4 times in the expansive path. At the final layer a 1 × 1 convolution is used to map each output feature vector to the desired number of classes. In addition, in order to ensure the size of input images and output images are same, this paper uses the U-Net network with padding, and the padding value is 1 [27].

After pixel prediction, the parameters of each γ’ precipitate can be calculated based on the pixel size.

## 4. Dataset and Training

There are 17 large-area SEM images under initial condition and different test conditions. These large-area SEM images with the size of 32 K × 32 K pixels are used as the dataset to train and test. Before running the program for image segmentation, each large-area SEM image is cut and resized to 512 × 512 pixels for ease of computation. These cut images as inputs are split into training (80%) and testing (20%) data sets. The algorithm is executed based on the open code of the U-Net [28].

The input images and their corresponding segmentation maps are used to train the U-Net model. In this model, the loss function of softmax cross-entropy loss is selected, which combines the softmax function with the cross entropy loss function [29,30]. The softmax function is defined as Equation (3) to calculate the predicted class score at each pixel position. Then the cross entropy is used to penalize the deviation of predicted class scores from the true class scores at all pixel positions, which is expressed as Equation (4). The training process is to obtain the appropriate model parameters by minimizing the loss function.

(3) $$p_{i}(x)=\frac{\exp(a_{i}(x))}{\sum_{i^{\prime }=1}^{K}\exp(a_{i^{\prime }}(x))}$$

(4) $$L=\sum_{x\in \Omega }\omega (x)\times \left[-\sum_{i=1}^{K}l_{i}(x)\log(p_{i}(x))\right]=-\sum_{x\in \Omega }\omega (x)\log(p_{l(x)}(x))$$

where *a_i_*(*x*) denotes the activation in feature channel *i* at the pixel position *x*, *x*∈Ω. *K* is the number of classes, *p_i_*(*x*) is the predicted class score of pixel *x* belong to *i*-th category. $\sum_{i=1}^{K}p_{i}\left(x\right)=1$. *l_i_* is the true class score of pixel *x* belong to *i*-th category. If pixel *x* belongs to *i*-th category, *l_i_* = 1 and *l_n_* = 0 for *n* ≠ *i*. Therefore, $-\sum_{i=1}^{K}l_{i}\left(x\right)\log p_{i}\left(x\right)=-\log\left(p_{l\left(x\right)}\left(x\right)\right)$, *l*(*x*) is the true label of the pixel *x*. ω(x) is a weight of pixel *x*, which is introduced to give the different importance for each pixel.

**Figure 2:**
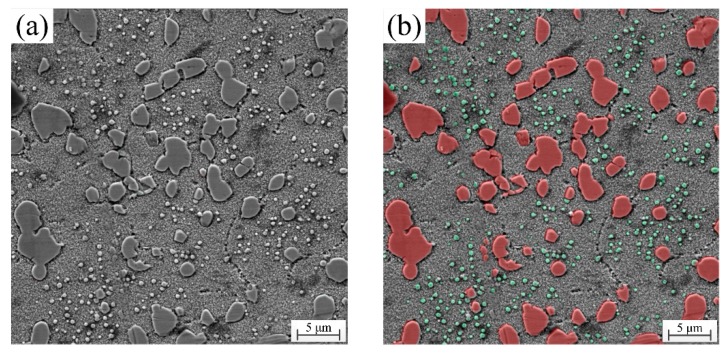
The original image (**a**) and segmented image (**b**) by the U-Net.

The segmented image by the U-Net model is shown in Figure 2. Red particles are primary γ’ precipitates, and green particles are secondary γ’ precipitates. Other areas are fine tertiary γ’ precipitates and γ matrix. Figure 2 shows that primary and secondary γ’ precipitates have been segmented accurately. The training accuracy is evaluated quantitatively by pixel accuracy (PA) [31], which computes the proportion of the correctly classified pixels to the total pixels. The calculate equation is expressed as Equation (5). The training process gives a high accuracy of 92.19%, and then the trained model is used to test.

(5) $$PA=\frac{\sum_{i=1}^{K}n_{ii}}{\sum_{i=1}^{K}t_{i}}$$

where *K* denotes the total number of categories in the image dataset, *t_i_* denotes the total number of pixels belongs to *i*-th category, and *n_ii_* denotes the number of pixels that belong to *i*-th category, and correctly predict as *i*-th category.

The U-Net algorithm is not applied to segment fine tertiary γ’ precipitates, because their distribution is too dense and the size is too small, leading low prediction accuracy. Therefore, the average sizes of tertiary γ’ precipitates are calculated after labeling of 1000 tertiary γ’ precipitates.

## 5. Results and Discussion

### 5.1. Initial γ’ Morphology

**Figure 3:**
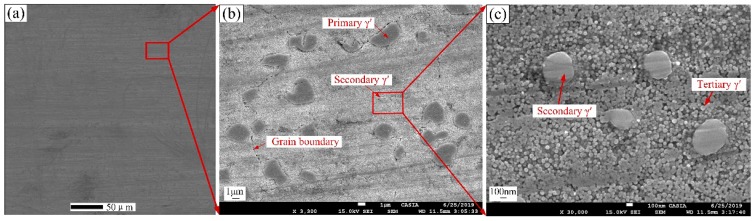
SEM image of initial γ’ morphology; (**a**) large-area SEM image of 32 K × 32 K pixels; (**b**) partly enlarged view of (**a**); (**c**) partly enlarged view of (**b**).

The SEM images of initial γ’ morphology are shown in Figure 3. Figure 3a is the large-area SEM image of 32 K × 32 K pixels, and Figure 3b,c are the partly enlarged images. GH4720Li superalloy is composed of matrix (γ phase) and multiple generations of γ’ precipitates. The γ’ precipitates include irregular primary γ’ precipitates, sphere-like secondary γ’ precipitates and spherical tertiary γ’ precipitates. The primary γ’ precipitates which remain undissolved during the solutioning stage are mainly distributed on the grain boundaries. Secondary and tertiary γ’ precipitates which are formed during the cooling process after solution treatment are distributed inside grains. The distribution of secondary γ’ precipitates is sparse and inhomogeneous, while the distribution of fine tertiary γ’ precipitates is dense and relatively uniform. The total volume fraction of multiple generations of γ’ precipitates in the alloy is about 45%.

### 5.2. Morphology Evolution of γ’ Precipitates

**Figure 4:**
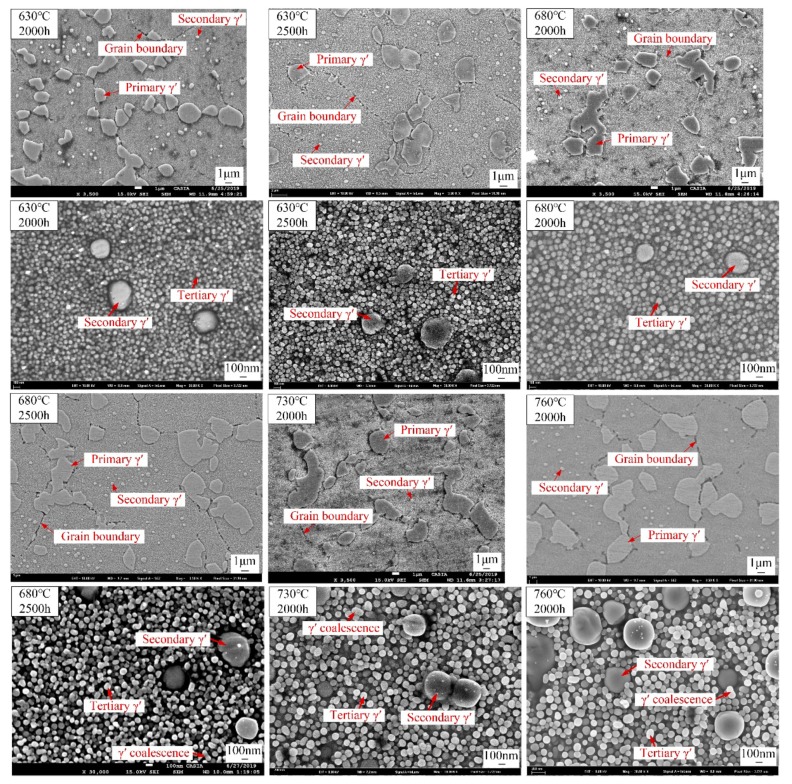
The γ’ morphology of the alloy under different thermal exposure conditions.

After long-term exposure for 2000 h and 2500 h at different temperatures, the γ’ morphologies are shown in Figure 4. The morphology and distribution of primary and secondary γ’ precipitates are not changed significantly, because the temperature is not high enough for the dissolution of γ’ precipitates and the long-range diffusion of alloying elements. The shape of secondary and tertiary γ’ precipitates is always spherical-like during thermal exposure, because the isotropic interfacial energy dominates over the anisotropic elastic strain energy, which contributes to maintain nearly spherical shapes. Tertiary γ’ precipitates coarsen after long-term thermal exposure because of the short-range diffusion of alloying elements at high temperature. The shape of some tertiary γ’ precipitates changes from spherical to ellipsoidal during γ’ growth, and a small amount of tertiary γ’ precipitates coalesce after 2500 h at 680 °C and after 2000 h at 730 °C, 760 °C.

### 5.3. Variations of Average Diameter and Area Fraction of γ’ Precipitates

**Figure 5:**
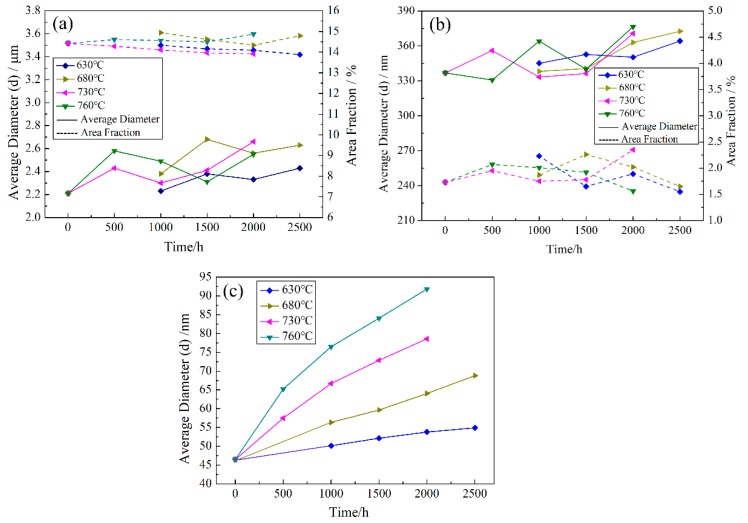
The variations of average diameter and area fraction of primary (**a**), secondary (**b**) and tertiary (**c**) γ’ precipitates during thermal exposure.

The average diameters and area fractions of primary, secondary and tertiary γ’ precipitates are calculated after precipitate segmentation by the U-Net model. Figure 5 presents parameter variations of different generations of γ’ precipitates during thermal exposure at different temperatures. The average diameters and area fractions of primary and secondary γ’ precipitates are almost constant during thermal exposure. The average diameter and area fraction of primary γ’ precipitates are about 2.4 μm and 14.5% respectively, as shown in Figure 5a. The corresponding parameters of secondary γ’ precipitates are about 350 nm and 2% respectively, as shown in Figure 5b. The average diameters of tertiary γ’ precipitates gradually increase with increasing thermal exposure time and temperature, and the shape of average diameter-time curves is similar to the parabolic shape, as shown in Figure 5c. The coarsening rate of tertiary γ’ precipitates increases first and then decreases with increasing time. Tertiary γ’ precipitates coarsen because the two-phase system is not in its lowest energy state at high temperatures due to the energy associated with γ’/γ interfaces. In such case, Ostwald ripening takes place to decrease the total energy of system by decreasing interfacial energy. The area fraction of tertiary γ’ precipitates is difficult to calculate accurately, because the distribution of tertiary γ’ precipitates is very dense, and it is difficult to separate one layer for accurate statistics. Moreover, there will be no significant change in area fraction of tertiary γ’ precipitates, because tertiary γ’ precipitates do not coarsen severely. Therefore, the variation of area fraction of tertiary γ’ precipitates is not discussed. The area fraction of tertiary γ’ precipitates before thermal exposure is estimated to be 30% by labeling a lot of tertiary γ’ precipitates in the SEM image artificially, and the labeled area is 5 μm × 5 μm.

### 5.4. Nanoindentation Hardness

**Figure 6:**
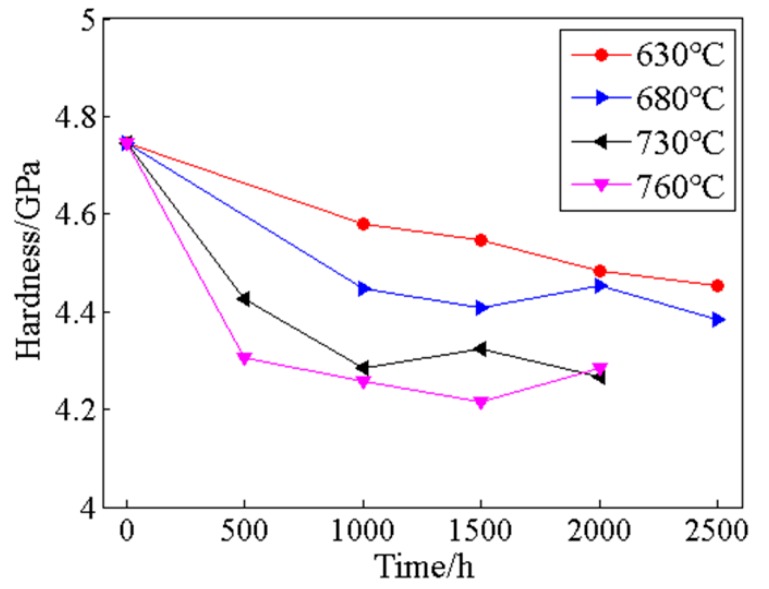
The variations of the average nanoindentation hardness of alloy during thermal exposure.

Hardness as an important property of alloys, which can determine many technological applications of alloys. The nanoindentation hardness of the γ’/γ coherent structures of the alloy was tested, and the variations of the average nanoindentation hardness during thermal exposure are shown in Figure 6. The hardness decreases with increasing thermal exposure temperature and time, and the change rate of hardness decreases with the increase of thermal exposure time. The reason is that hardness is highly sensitive to the microstructure of the alloy. During long-term thermal exposure, there is no obvious change in the secondary γ’ precipitates in the interior of grains. Therefore, the hardness variation of the γ’/γ coherent structures of the ally is caused by the coarsening of tertiary γ’ precipitates.

## 6. Modeling the Coarsening Kinetics of γ’ Precipitates

The classic Lifshitz–Slyozov–Wagner (LSW) model [32,33] is the main approach to predict γ’ coarsening, but this model is strictly applicable when the volume fraction f of γ’ precipitates is small and approaches zero. Then the Lifshitz–Slyozov encounter modified (LSEM) model was developed with the assumption of instantaneous coalescence of γ’ particles, and this model removed the assumption of f = 0 [34]. The LSEM model also describes a linear relationship between the cube of average precipitate radius and the holding time at a high temperature, which express the coarsening process of γ’ precipitate is controlled by solute diffusion through the matrix. The expression of the LSEM model is given by

(6) $$r_{t}^{3}-r_{0}^{3}=K_{L}t$$

where *t* is the thermal exposure time, *r_t_* and *r*_0_ are the average radius of γ’ precipitates at time *t* and initial time, respectively. *K_L_* is the coarsening rate constant of the LSEM model, which can be calculated by equation as following

(7) $$K_{L}=\frac{6\sigma V_{m}c_{e}D{\bar{r}}^{3}}{RT\gamma }$$

where *σ* is the surface energy associated with the precipitate-matrix interface, *V_m_* is the molar volume of γ’ precipitates, *c_e_* is the per mole volume fraction of solute in equilibrium, *D* is the temperature-dependent diffusion coefficient. $\bar{r}=r-r_{c}$, where *r* is the average precipitate radius, and *r_c_* is the critical radius. *R* is the gas constant, *T* is the thermodynamic temperature, *γ* is a constant factor. The values of parameters $\bar{r}$ and γ are determined by the given volume fraction of γ’ precipitates.

**Figure 7:**
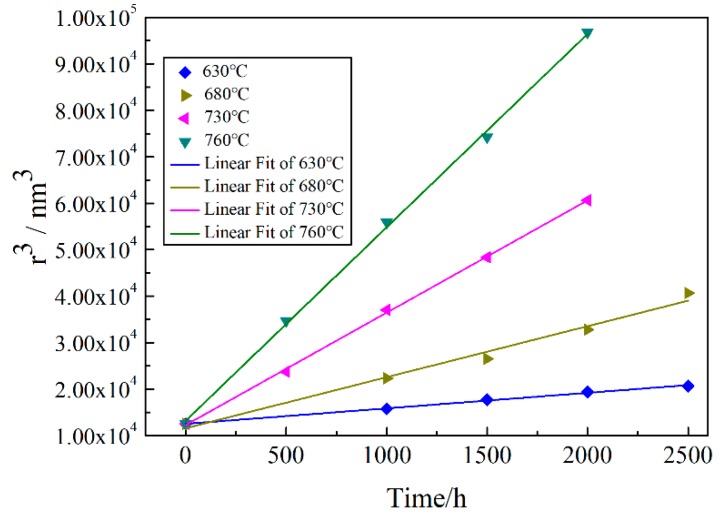
The linear fit plots of *r*^3^ vs. *t* of the tertiary γ’ precipitates.

The LSEM model is applied to modeling the coarsening kinetics of tertiary γ’ precipitates during long-term thermal exposure. Figure 7 shows the linear relationship between the cube of average radius of tertiary γ’ precipitates and the thermal exposure time at temperature ranging from 630 to 760 °C. The coarsening rate *K_L_* is 3.33 nm^3^/h at 630 °C, 10.97 nm^3^/h at 680 °C, 24.17 nm^3^/h at 730 °C and 41.61 nm^3^/h at 760 °C. The corresponding fitting parameters *R*^2^ are 0.9956, 0.9805, 0.9992 and 0.9987, respectively, which also proves the growth of tertiary γ’ precipitate is consistent with the matrix-diffusion-controlled coarsening mechanism.

## 7. A Microstructure-Related Hardness Model

During the thermal exposure, the variations of hardness are caused by the γ’ coarsening in the alloy. The overall nanoindentation hardness of alloys can be divided into two parts [35]. One is the matrix hardness with the contribution by other sources rather than γ’ precipitates, including solid-solution strengthening, grain boundary strengthening. The contribution of grain boundary strengthening can be ignored because all indentations are made at the interior of grains. The other is the hardness due to the precipitation strengthening. By using Tabor’s empirical relationship Hppt = 3σppt [18,36] and σppt = Mτppt [1], where M is the Taylor factor, which is equal to three for polycrystalline fcc-base alloys, the contributions of precipitation hardening are estimated in terms of the critical resolved shear stress (CRSS). The overall hardness of the alloy is expressed as Equation (8).

(8) $$H_{tot}=H_{mat}+H_{ppt}=H_{mat}+9\times \tau_{ppt}$$

where *H_tot_* is the overall hardness of the γ’/γ coherent structures, *H_mat_* is the hardness which includes the inherent hardness of the γ matrix and the solid-solution strengthening contribution. *H_ppt_* is the hardness due to the precipitation strengthening contribution. *τ_ppt_* is the CRSS of the bimodal particle system, including secondary and tertiary γ’ precipitates.

For the small γ’ precipitates, the classic precipitation strengthening models are based on the fact that dislocations pair-up to cut through the γ’ precipitates. The leading dislocation cut through the γ’ precipitates and creates the anti-phase boundary, then the trailing dislocation glides in the same plane to remove it. The size and distribution of γ’ precipitates affect the precipitation strengthening by strong coupling and by weak coupling [37,38], and it is traditionally assumed that the maximum particle strength occurs when the strong and weak pair-coupling models converge at a constant temperature. The strong and weak-pair coupling configurations are dictated when *r > r_m_* and *r < r_m_*, respectively. *r_m_* is the precipitate radius with maximum strength. The corresponding CRSS from these two mechanisms is expressed as Equations (9) and (10) respectively.

(9) $$\tau_{strong}=0.5\times 0.72(\frac{G^{\gamma^{\prime }}b}{2r})f^{0.5}{\left(\frac{2\pi r\gamma_{APB}}{G^{\gamma^{\prime }}b^{2}}-1\right)}^{0.5}$$

(10) $$\tau_{weak}=\frac{\gamma_{APB}}{2b}\left[{\left(\frac{6\gamma_{APB}fr}{\pi G^{\gamma^{\prime }}b^{2}}\right)}^{0.5}-f\right]$$

where G^γ’^ is the shear modulus of γ’ precipitates, and the value is 85 GPa. *b* is the Burgers vector, and the value is 0.248 nm. *r* and *f* are the average radius and volume fraction of γ’ precipitates, and it is assumed that the volume fraction of γ’ precipitates is equal to their area fraction during calculation. *γ_APB_* is the APB energy, and the value is 0.29 J/m^2^ for GH4720Li alloy [1]. When the inter-particle spacing and the size of γ’ precipitates are sufficiently large, the Orowan mechanism can occur [2]. However, it is generally difficult to observe the Orowan looping in polycrystalline Ni-based superalloys [39,40]. Therefore, the CRSS of the Orowan mechanism is not considered for estimating the precipitation strengthening in this paper.

**Figure 8:**
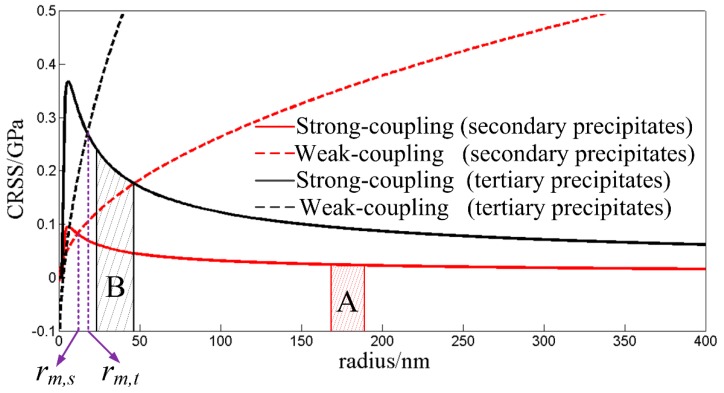
Relationship between the critical resolved shear stress (CRSS) of secondary and tertiary γ’ precipitates and the mean γ’ radius.

In order to calculate the CRSS of the bimodal particle system, the bimodal γ’ size distributions are translated into two types of unimodal γ’ size distribution. In this case, the CRSS of secondary and tertiary γ’ precipitates can be calculated respectively as individual unimodal γ’ size distribution using Equations (9) and (10). The CRSS of the bimodal particle system is assumed to be the simple summation of CRSS of these two types of unimodal γ’ size distribution, *τ_ppt_* = *τ_ppt,s_* + *τ_ppt,t_*. *τ_ppt,s_* is the CRSS of secondary γ’ precipitates, and *τ_ppt,t_* is the CRSS of tertiary γ’ precipitates. Figure 8 shows the relationship between the CRSS of secondary and tertiary γ’ precipitates and the mean radius of γ’ precipitates. *r_m,s_* is the secondary precipitate radius with maximum strength, and *r_m,t_* is the tertiary precipitate radius with maximum strength. The regions A and B represent the distribution range of mean radius of the secondary and tertiary γ’ precipitates respectively during long-term thermal exposure. It could be seen that mean radius of secondary γ’ precipitates is larger than *r_m,s_*, and mean radius of tertiary γ’ precipitates is larger than *r_m,t_*. Therefore, the precipitation strengthening in the bimodal particle system follows the strong coupling mechanism. The CRSS of secondary and tertiary γ’ precipitates which will be calculated by Equation (9) decreases with increasing γ’ radius, so the corresponding hardness decreases during γ’ coarsening. The microstructure-related hardness model of GH4720Li alloy with multiple generations of γ’ precipitates can be established by inserting Equation (9) into Equation (8), and its expression is given by Equation (11). This model can be used to predict the hardness of GH4720Li alloy with different microstructures. During the long-term thermal exposure at 630–760 °C, it is assumed that the area fractions of secondary and tertiary γ’ precipitates used in Equation (9) keep constant, and the values are 2% and 30% respectively.

(11) $$H_{tot}=H_{mat}+H_{ppt}=H_{mat}+9\times \tau_{ppt}=H_{mat}+9\times (\tau_{strong,s}+\tau_{strong,t})$$

**Figure 9:**
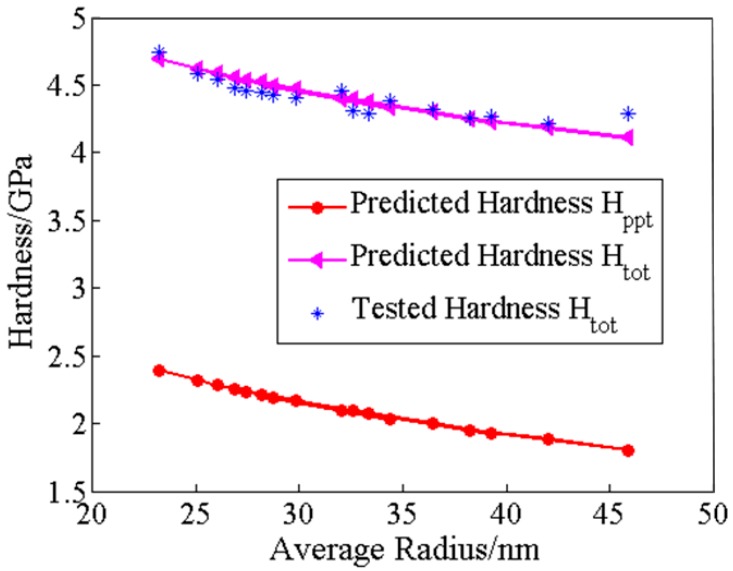
Relation between the nanoindentation hardness *H_tot_*, *H_ppt_* and the mean radius of tertiary γ’ precipitates.

Figure 9 shows the hardness variations during coarsening of tertiary γ’ precipitates. The overall hardness *H_tot_* includes the values predicted by above microstructure-related hardness model and tested by nanoindentation experiments. It could be seen that the microstructure-related hardness model can accurately predict the hardness of GH4720Li alloy during long-term thermal exposure. Similar trend of the hardness *H_tot_* and *H_ppt_* also indicates that the degradation of overall hardness of GH4720Li alloy is caused by the coarsening of γ’ predictions, and the hardness *H_mat_* is constant. Comparing the hardness values of *H_tot_* and *H_ppt_* at different mean radii of tertiary γ’ precipitates, the value of *H_mat_* is about 2.3 GPa.

## 8. Conclusions

In this paper, thermal exposure tests and microstructural detection were conducted to study the precipitate stability and the microstructure related hardness of a polycrystalline Ni-based superalloy GH4720Li during long-term thermal exposure at 630–760 °C. In order to accurately and automatically derive the morphological parameters of γ’ precipitates from extremely large-area SEM images with the size of 32 K × 32 K pixels (327.68 μm × 327.68 μm), a new strategy to segment γ’ precipitates by deep learning method is proposed. The deep learning method of U-Net was successfully applied to accurately and quickly segment different generations of γ’ precipitates with similar gray values in the large-area SEM images. As far as the authors’ knowledge, this is the first time the deep learning method has been applied to obtain the morphological parameters of γ’ precipitates for subsequent quantitative research, which can effectively promote the application of deep learning in material sciences.

Based on the experimental results, primary and secondary γ’ precipitates show good stability during long-term thermal exposure. On contrary, the sizes of tertiary γ’ precipitates increase significantly with increasing thermal exposure time and temperature, leading to the hardness degradation of the alloy. A microstructure-related hardness model is established, and it could accurately predict the hardness of GH4720Li alloy with different microstructures during thermal exposure. As far as our knowledge, it is the first time the microstructure-related hardness model has been used to correlate the γ’ morphology and the hardness of Ni-based alloys.

## Figures and Tables

**Table 1:** Experimental parameters of thermal exposure tests.

Temperature	Time				
	500 h	1000 h	1500 h	2000 h	2500 h
Initial	-	-	-	-	-
630 °C	-	√	√	√	√
680 °C	-	√	√	√	√
730 °C	√	√	√	√	-
760 °C	√	√	√	√	-
